# Design and developing a robot-assisted cell batch microinjection system for zebrafish embryo

**DOI:** 10.1038/s41378-024-00809-y

**Published:** 2025-02-20

**Authors:** Xiangyu Guo, Antian Zhao, Youchao Zhang, Huanyu Jiang, Longhua Tang, Bo Lu, Yibin Ying, Mingchuan Zhou

**Affiliations:** 1 Robotic Micro-nano Manipulation Lab, College of Biosystems Engineering and Food Science, Zhejiang University, Hangzhou, China; 2 College of Optical Science and Engineering, Zhejiang University, Hangzhou, China; 3 Robotics and Microsystems Center, School of Mechanical and Electric Engineering, Soochow University, Suzhou, China

**Keywords:** Electrical and electronic engineering, Optical sensors

## Abstract

The microinjection of Zebrafish embryos is significant to life science and biomedical research. In this article, a novel automated system is developed for cell microinjection. A sophisticated microfluidic chip is designed to transport, hold, and inject cells continuously. For the first time, a microinjector with microforce perception is proposed and integrated within the enclosed microfluidic chip to judge whether cells have been successfully punctured. The deep learning model is employed to detect the yolk center of zebrafish embryos and locate the position of the injection needle within the yolk, which enables enhancing the precision of cell injection. A prototype is fabricated to achieve automatic batch microinjection. Experimental results demonstrated that the injection efficiency is about 20 seconds per cell. Cell puncture success rate and cell survival rate are 100% and 84%, respectively. Compared to manual operation, this proposed system improves cell operation efficiency and cell survival rate. The proposed microinjection system has the potential to greatly reduce the workload of the experimenters and shorten the relevant study period.

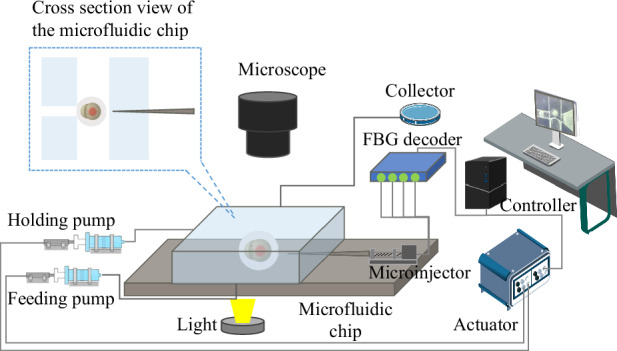

## Introduction

Exogenous material delivery is an indispensable biomedical research technique with widespread applications in gene editing, drug development, biological breeding, and multifunctional stem cell induction^1–4^. There are three approaches to delivering macromolecules into living cells: chemical methods (synthetic carriers), biological methods (viral vectors), and physical methods^5–7^. Chemical methods depend on the self-assembly of a liposome encapsulating the target molecules for delivery^8^. Subsequently, the liposome transports the enclosed macromolecules into the cell. These methods are simple and safe, but the low absorption rate of the cell membrane, the insufficient release of DNA molecules, and the lack of specificity to the target result in limited cell transfection efficiency. Biological methods mainly use viruses as carriers to construct delivery tools, which have the advantage of high efficiency^9^. However, their application scope is limited due to the specific targeting of cells. Physical methods refer to delivering exogenous substances into the cell by creating pores on the cell membrane. These methods include microinjection, electroporation, gene gun, and so on^10–12^. Although the gene gun is simple to operate, the necessary consumables add additional cost^13–15^, this method has a low conversion rate and a high mutation rate in offspring. The electroporation method utilizes high-voltage pulses to alter cell permeability for the entry of exogenous DNA into cells. However, these high-voltage pulses might damage the cells or even render them inactive^16–18^. Among these methods, microinjection is the only one to achieve quantitative and targeted injection of exogenous molecules. Unfortunately, operators must switch objectives frequently to find cells at low magnification and inject them under high magnification^19–22^. It limits the efficiency of cell manipulation.

To improve the efficiency of cell injection, the robot-assisted manipulation platform is developed to replace manual operation, which utilizes two robotic hands that imitate the operator’s actions to achieve cell manipulation. It significantly improves the efficiency of cell manipulation and the consistency of results. On this basis, Wang et al.^23^ proposed a pipelined batch operation of cell nuclear transplantation based on a micromanipulation system, which adds two micropipettes to transport and collect oocytes. Because of the microscale effect of the fluid, it is difficult to ensure that only one cell is exported at a time. So, Liu et al.^24^ developed a batch cell nuclear transfer based on a microfluidic groove, which moves the carrier platform to realize the sequential injection of cells. In addition, Wang et al.^25,26^ proposed an array cell automatic injection system based on microfluidic chips, which realized continuous injection of cells through the cooperative movement of microneedles and chips under visual feedback. However, the cells need to be put into the groove one by one in advance, which increases the operation time of the task. Andrea et al.^27^ reported a microfluidic-based approach for single-cell microinjection in which fluid streams direct a cell onto a fixed microneedle. The cells are separated from the needle by pneumatic valves and fluid movements. In addition, Chow et al.^28^ proposed a high-throughput injection system for human small cells, which could efficiently inject cells. However, how to efficiently place and recycle cells is a challenge that needs to be solved. To solve this challenge, Arash et al.^29^ proposed a microfluidic system for microinjection, which uses flexible and compliant channels and electroosmosis. The system provides cell transportation and collection functions. However, the proposed electroosmosis method may cause exogenous substances’ properties to be changed. It is undeniable that the development of microfluidic chip technology has brought the dawn of high-throughput cell injection^30–32^. At present, visual perception is the main source of information to judge the state of cell puncture in microfluidic chips. The utilization of only visual information is unreliable, and it is a challenging task to integrate force sensors in the narrow microfluidic space to enhance cell state perception inside the microfluidic chip.

In response to these points, a novel automated microinjection system is developed based on a microfluidic chip and integrated microinjector with force-sensing in its narrow space for the first time. The proposed microinjection system greatly reduces the workload of the experimenters and shortens the relevant study period. The features and contributions of this work are listed as follows:

- An automated microinjection system is developed based on a microfluidic chip with a sophisticated structure to transport, hold, inject, and collect cells continuously. It does not require frequent switching of the objective lens and preprocessing, which improves operational efficiency.
- A microinjector with microforce sensing is proposed and integrated into a narrow microfluidic chip for the first time. The deep learning model is employed to detect the yolk center of zebrafish embryos and locate the position of the injection needle within the yolk.
- A prototype is fabricated to achieve automatic batch microinjection. Experimental results demonstrated injection efficiency is 20 s per cell. Cell puncture success rate and cell survival rate are 100% and 84%, respectively.

The remainder of the article is organized as follows. First, the experimental results are introduced and discussed. Then, a conclusion summarizes the paper and presents future work. Finally, the design and manufacture of the automatic microinjection system are described in detail.

## Materials and methods

### Design and development of microinjection system prototype

**Fig. 1:**
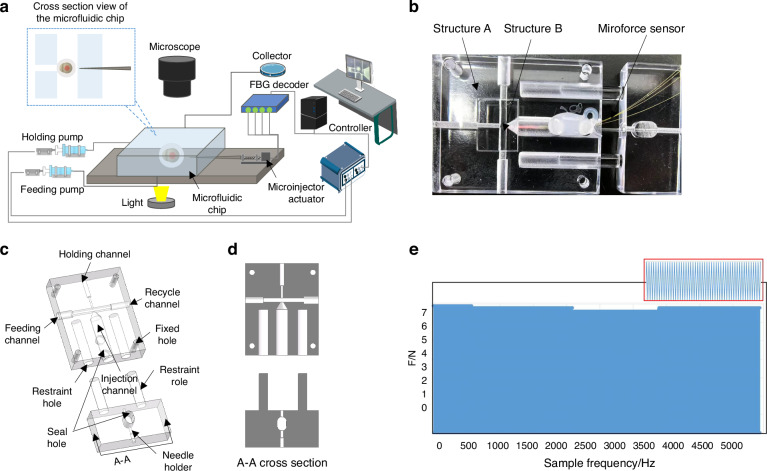
System setup. **a** Schematic diagram of microinjection system. The system is mainly composed of an injection module, a vision module, and a control module. The injection module consists of a microfluidic chip and a microsyringe with force sensing. The vision module and control module includes a computer, a microscopic camera, and a motion controller. **b** Microinjection chip. **c** Design details of microfluidic chip three-dimensional (3D) model. The microfluidic chip has four channels to connect to external devices. The right channel is connected to a microinjector for cell puncture and injection of foreign substances. The middle channel connects two micropumps for cell delivery and recovery. The left channel is connected to the suction pump for cell fixation. **d** A-A Cross-section of a microfluidic chip. **e** Fatigue test of sealant

**Fig. 2:**
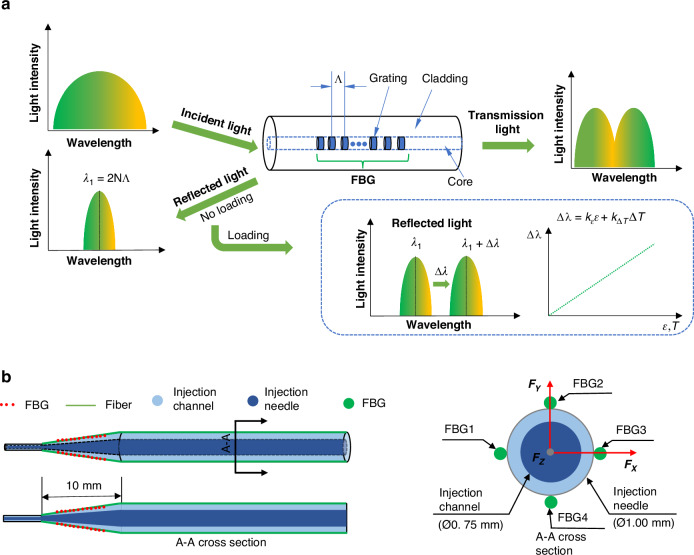
Schematic diagram of 3D FBG sensor. **a** Working principle of fiber Bragg grating. The sensing principle of fiber optic grating is based on the periodic modulation of the effective refractive index within the core of the optical fiber. **b** Schematic of the microinjection with microforce sensor. Four optical fibers are arranged in the external contour of the glass needle at 90°, which senses the wavelength shift with the needle deformation. Each optical fiber contains an FBG sensor with a length of 1 cm. The four optical fibers need to be glued close to the needle tip to be more sensitive to the deformation of the needle. The glass needle (outer diameter 1 *mm*) has a hollow channel for injecting exogenous substances

The automatic microinjection system is developed to inject cells, which consists of an injection module, visual module, and control module, as shown in Fig. 1a. The injection module includes a microinjector and a microfluidic chip. The injection needle (60 μm) is manufactured by a micropipette puller (P-1000, Sutter Instrument Co., USA), which is used to heat and pull a glass micropipette with an external diameter of 1.0 mm and an internal diameter of 0.75 mm. The injection needle is integrated with a self-developed micro-force sensor with more details in Fig. 2, which injects exogenous substances into cells while detecting cellular mechanical information.

The microfluidic chip is shown in Fig. 1b–e. The microfluidic chip consists of structures A and B, which are manufactured by stereo lithography appearance. Structure B is attached by gluing to the groove of structure A. The microfluidic chip has four channels to connect to external devices. The chip size is 30 mm in length and 20 mm in width. Cells are injected from a 2.2 mm diameter feeding channel and passed through a 1.5 mm diameter internal channel into the recycle channel. The internal diameter of the holding channel is used to hold the cells with 0.75 mm diameter. One end of the glass needle is fixed to the needle holder by medical adhesive, and the other end is fixed to the injection channel by the flexible sealant (silicone). The flexible sealant has good sealing and elastic properties. The restraint hole and the restraint rod are designed to ensure the linear movement of the injection needle. The injection channel and the microinjector are fixed by a flexible material, which ensures that the chip is sealed and the injection needle has 1 mm displacement puncture cells. The feeding channel connects one micropump for cell transportation. The holding channel is connected to the negative pressure pump for cell holding. The recycle channel is directly connected to the petri dish for cell collection.

For the visual module, the microscope camera (HAYEAR, Shenzhen Haiyue Electronics Co., Ltd, China) is applied to detect the cell status and track the position of cells, which can be used to control the movement of actuators based on visual information. The control module consists of a computer, actuator (DM420S, Beijing Haijie Jiachuang Tech. Ltd., China) with a sampling frequency of 4 kHz, and a fiber Bragg grating (FBG) decoder with 1000 Hz sampling frequency (Tv-1600, Beijing Tongwei Technology Co., Ltd, China).

### Design and manufacture of microinjection with force sensor

The internal space of microfluidic chips is narrow, which makes it difficult to install other force sensors to detect the state of cells. The mainstream method extracts cell deformation features through the visual system to determine whether the cell has been successfully pierced. Compared with the mechanical jump signal, the former is unreliable. Therefore, a novel micro-force sensor has been proposed for detecting the state of cells inside microfluidic chips. The proposed sensor can be well integrated with the microneedle, which has a compact structure and can be easily installed in the microfluidic chip. The developed sensor sets a refractive index periodic distribution grating area at a specific position of the optical fiber, and the light waves of a specific wavelength (Bragg reflection light) will be reflected in this area, resulting in a wavelength shift. The developed sensor mainly monitors the axial force of cells, and specific design details are shown in Fig. 2.

Four optical fibers are arranged in the external contour of the glass needle at 90°, which senses the wavelength shift with the needle deformation. Each optical fiber contains an FBG sensor with a length of 1 cm. The four optical fibers need to be glued close to the needle tip to be more sensitive to the deformation of the needle. The glass needle (outer diameter 1 mm) has a hollow channel for injecting exogenous substances. As shown in Fig. 2a, the sensing principle of fiber optic grating is based on the periodic modulation of the effective refractive index within the core of the optical fiber. The relationship between the central wavelength of the fiber grating *λ* and the refractive index *N* is

1 $$\lambda =2N\,\cdot \,\Lambda$$

where *Λ* is the periodicity of the grating. The shift in Bragg wavelength of the FBG sensors is linearly dependent on local strain and temperature change. The wavelength shift Δ*λ* can be calculated as

2 $$\Delta \lambda ={k}_{\varepsilon }\varepsilon +{k}_{\Delta T}\Delta T$$

where *k*_*ε*_ and *k*_Δ*T*_ are the constant coefficients, *ε* is the strain of the FBG, and Δ*T* is the temperature change.

#### Transverse force calculation

The strain of the fiber grating *ε* is calculated as

3 $$\varepsilon =\frac{{F}_{i}d}{EI}\gamma$$

where *E*, *γ*, and *I* represent Young’s modulus, the inertia torque, and the radius of the tube, respectively. *F*_*i*_ is the force applied to the needle tip, and *d* is the distance between the needle tip and the FBG sensor.

To eliminate the common mode caused by the temperature change and axial force of the three sensors, it is solved by subtracting the Bragg mean value, and the processed differential mode signal is expressed by the linear formula:

4 $$\begin{array}{ll}\Delta {s}_{i}=\Delta {\lambda }_{i}-\Delta {\lambda }_{{\rm {mean}}}\\\qquad ={k}_{\varepsilon i}{\varepsilon }_{i}-\displaystyle\frac{1}{3}\mathop{\sum }\limits_{i=1}^{3}{k}_{\varepsilon i}{\varepsilon }_{i},\,\,i=1,2,3\end{array}$$

where Δ*s*_*i*_ and Δ*λ*_*i*_ are the relative wavelength change and the wavelength shift of the FBG sensor *i*, respectively. Δ*λ*_mean_ represents the average value of the wavelength drift of the three optical fibers. So, transverse force *F*_T_ is given by

5 $$\begin{array}{c}{F}_{{\rm {T}}}={C}_{{\rm {tran}}}\Delta {S}_{i}\end{array}=\left[\begin{array}{ccc}{C}_{11}&{C}_{12}&{C}_{13}\\ {C}_{21}&{C}_{22}&{C}_{23}\\\end{array}\right]\left[\begin{array}{c}\Delta {s}_{1}\\ \Delta {s}_{2}\\ \Delta {s}_{3}\end{array}\right]$$

where *F*_T_ = [*F*_*X*_, *F*_*Y*_], Δ*S*_*i*_ = [Δ*s*_1_, Δ*s*_2_, Δ*s*_3_], *C*_tran_ is a 2 × 3 coefficient matrix representing the linear mapping from the FBG sensor readings to the transverse forces.

#### Axial force calculation

To measure the axial puncture force of cells, four FBG optical fibers are employed. The relationship between axial force *F*_*Z*_ and wavelength shift Δ*λ*_*i*_ is

6 $$\begin{array}{ll}{F}_{Z}=\begin{array}{c}{A}_{{\rm {axi}}}\Delta {\lambda }_{i}\end{array}\\\qquad \,=\left[\begin{array}{cccc}{A}_{11}&{A}_{12}&{A}_{13}&{A}_{14}\end{array}\right]\left[\begin{array}{c}\Delta {\lambda }_{1}\\ \Delta {\lambda }_{2}\\ \Delta {\lambda }_{3}\\ \Delta {\lambda }_{4}\end{array}\right]\end{array}$$

#### Force calibration

**Fig. 3:**
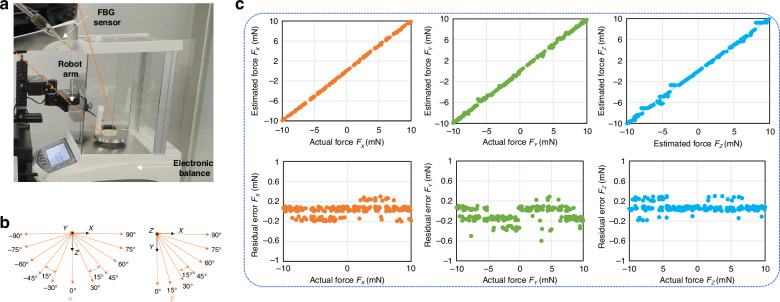
FBG force sensor calibration. **a** Microinjector with a 3D force sensor calibration platform. **b** View of variation of pitch angle *α* and *β*. **c** Diagram of Linear fitting and residual for three-dimensional force. The actual force and estimated force are fitted linearly, and the error between the actual force and estimated force is calculated. The fitting slope between the actual force and the calculated force *F*_*X*_, *F*_*Y*_, and *F*_*Z*_ are close to 1

To achieve more accurate cell force sensing, the designed sensor is calibrated to eliminate errors during manufacturing and integration processes. The calibration platform is illustrated in Fig. 3a–b.

The calibration system consists of a robotic arm, an electronic balance, a weight, an FBG sensor decoder, a computer, and a monitor. During the calibration procedure, the pitch angle *α* ranged from (−30° to −90°) to (30°–90°) with 15° intervals, and the roll angle *β* ranged from 0° to 90° with 15° intervals. The force magnitude ranges from 0 to 10 mN. A total of 350 samples were collected.

The relationship between *F* and the total calibration force *F*_P_ is

7 $$\begin{array}{ll}F=\left[\begin{array}{ccc}{F}_{X}&{F}_{Y}&{F}_{Z}\\ \end{array}\right]\\\quad \,=\left[\begin{array}{ccc}{F}_{{\rm {P}}}\sin \alpha \cos \beta &{F}_{{\rm {P}}}\sin \alpha \sin \beta &{F}_{Z}\cos \alpha \end{array}\right]\quad \end{array}$$

Based on the correction data, the optimal-fit pseudoinverse matrix of matrix *C*_tran_ and *A*_axi_ are calculated with the least-square method:

8 $$\begin{array}{ll}{C}_{{ {tran}}}\,=\,\left[\begin{array}{ccc}-0.025&0.111&-0.086\\ -0.143&0.274&-0.131\\ \end{array}\right]\end{array}$$

9 $${A}_{{ {axi}}}=\left[\begin{array}{cccc}-0.167&0.109&-0.094&0.382\\ \end{array}\right]$$

Its transverse force and axial force resolution are about 0.274 and 0.382 mN, respectively, and the linear fitting results are depicted in Fig. 3c. The fitting slope between the actual force and the calculated force *F*_*X*_, *F*_*Y*_, and *F*_*Z*_ are close to 1. The samples between −10 and 10 mN are divided into 10 samples with an interval of 1 mN, and the residual error is distributed within the range of −0.60 to 0.40 mN. The average error of *F*_*X*_, *F*_*Y*_, and *F*_*Z*_ are 0.096, 0.136, and 0.068 mN, respectively.

### Cell manipulation strategy based on visual and force perception

**Fig. 4:**
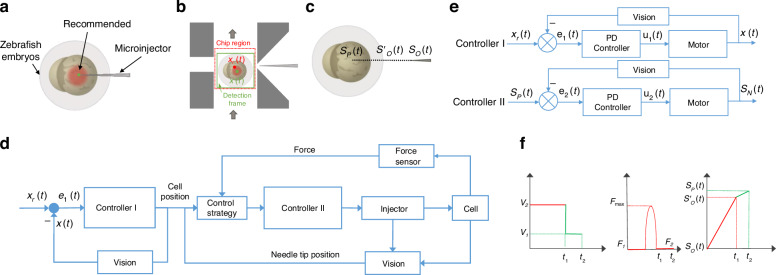
Control system. **a** Schematic diagram of the recommended puncture site for zebrafish embryos. **b** Visual detection of zebrafish embryo inside the microfluidic chip. When the cell enters the visual detection region inside the microfluidic chip, the YOLOX algorithm can detect the cell’s position and the yolk’s centroid. **c** Diagram of the microinjector from the initial location to the cell’s centroid. **d** Schematic diagram of the algorithm structure of vision and force fusion control cell injection. **e** Algorithm structure of controller I and controller II. **f** Speed, force, and position during puncture

During injection, foreign substances are injected into the yolk center of the zebrafish embryo (Fig. 4a). To ensure the operational effect, visual information and force information are fused to control the position of the microinjection (Fig. 4b–f). The complete injection process of a single cell can be decomposed into four tasks:

*Cell transportation*: The cell is transported into the microfluidic channel by a micropump. The cell is transported by fluid into the field of view of the microscope, which is detected by the YOLOX network^33^ and computes the centroid position *x*(*t*) of the cell. Based on the position information provided by visual feedback, the cells are transported to the target position *x*_r_(*t*) by a proportional differential (PD) algorithm driven by the fluid. The algorithm is

10 $${u}_{1}(t)={K}_{{ {p}}1}{e}_{1}(t)+{K}_{ {{d}}1}\frac{{\rm {d}}{e}_{1}(t)}{{\rm {d}}t}$$

11 $${e}_{1}(t)={x}_{{\rm {r}}}(t)-x(t)$$

where *K*_p1_ is the proportional gain, and *K*_*d*1_ is the derivative gain. *u*_1_(*t*) is the pulse signal, which is output by the PD controller. *e*_1_(*t*) is the difference between the expected position and the actual position of the cell.

*Cell holding*: The cell is held and fixed by a micro vacuum pump when it is detected near the holding channel within the microscopic field of view.

*Cell puncture*: After the cells are held, the microinjection starts to puncture the cells. When the injection needle contacts the cell, the sensor is stressed. The sensor detects the maximum stress just before the cell is about to be punctured. When the cell is punctured, the stress detected by the sensor suddenly disappears. Therefore, the signal of the force sensor is detected to determine whether the cell puncture is successful. The cell membrane is successfully pierced with a step signal from the force sensor. Tip position and force sensor status are:

12 $$F(t)=\left\{\begin{array}{ll}{F}_{1}\quad\,{S}_{{\rm {N}}}(t)\in [{S}_{{\rm {N}}},{S}_{{\rm {O}}}^{{\prime} })\\ {F}_{{\rm {max}}}\quad{S}_{{\rm {N}}}(t)\,{\text{in}}\,{S}_{{\rm {O}}}^{{\prime} }\\ {F}_{2}\quad{S}_{{\rm {N}}}(t)\in ({S}_{{\rm {O}}}^{{\prime} },{S}_{{\rm {P}}}]\text{}\end{array}\right.$$

Then, the puncture speed of the microinjector changes from *V*_2_ to *V*_1_, which further punctures the center of the yolk based on the PD algorithm. After the needle tip reaches the desired position, the external material is injected into the cell using a microinjection pump. The speed changes based on

13 $$V(t)=\left\{\begin{array}{ll}{V}_{2}\quad {S}_{{ {N}}}(t)\in [{S}_{{ {O}}}(t),{S}_{{ {O}}}^{{\prime} }(t)]\\ {V}_{1}\quad {S}_{{ {N}}}(t)\in [{S}_{{ {O}}}^{{\prime} }(t),{S}_{{ {P}}}(t)]\end{array}\right.$$

where *S*_*N*_(*t*) is the position of the needle tip at time *t*, and *S*_*O*_(*t*) is the position of the needle tip at the initial moment. ${S}_{\rm {{O}}}^{{\prime} }(t)$ represents the position of the cell pierced, and *S*_*P*_(*t*) represents the center of the yolk. The MMPOSE network is employed to detect the needle tip position^34^.

The needle tip position control strategy is

14 $${u}_{2}(t)={K}_{{ {p}}2}{e}_{2}(t)+{K}_{{ {d}}2}\frac{{ {d}}{e}_{2}(t)}{{ {d}}t}$$

15 $${e}_{2}(t)={S}_{{ {P}}}(t)-{S}_{{ {N}}}(t)$$

where *K*_p2_ is the proportional gain, and *K*_*d*2_ is the derivative gain. *u*_2_(*t*) is the output signal of the PD controller, and *e*_2_(*t*) is the difference between the expected position and the actual position of the cell.

*Cell recycling*: the injected cell is recovered into a culture dish using a microinjection pump.

### Cell manipulation parameter

**Fig. 5:**
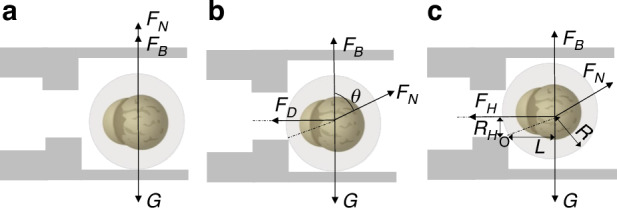
Schematic diagram of cell mechanics analysis. **a** Cell initial state in the microfluidic chip. **b** Cell minimum drag force analysis in the microfluidic chip. **c** Cell minimum holding force analysis in the microfluidic chip

When a cell is in a stationary state within a microfluidic channel (Fig. 5a), the cell is affected by the gravity *G*, the buoyancy force *F*_B_, and the normal force F_*N*_. The relationship is given by

16 $${F}_{{\rm {N}}}=G-{F}_{{\rm {B}}}=\frac{4}{3}({\rho }_{{\rm {c}}}-{\rho }_{f})\pi {R}^{3}g$$

where *ρ*_c_ and *ρ*_f_ are the density of the cell and the fluid, respectively. *R* is the radius of the cell, and *g* is the gravitational acceleration. As shown in Fig. 5b, the minimum drag force *F*_D_ can be calculated as

17 $$G-{F}_{{\rm {B}}}={F}_{{\rm {N}}}\times \cos \theta$$

18 $${F}_{{\rm {D}}}={F}_{{\rm {N}}}\times \sin \theta$$

When the cell is held, the force analysis is shown in Fig. 5c. The relationship can be calculated as

19 $${M}_{{F}_{{\rm {B}}}}+{M}_{{F}_{{\rm {N}}}}+{M}_{{\rm {G}}}+{M}_{{F}_{{\rm {H}}}}=0$$

where ${M}_{{F}_{{\rm {B}}}}$, ${M}_{{F}_{{\rm {N}}}}$, ${M}_{{F}_{{\rm {G}}}}$, and ${M}_{{F}_{{\rm {H}}}}$ are the moments of the *F*_B_, *F*_*N*_, *F*_G_, and *F*_H_ to the point *O*, respectively. The contact force *F*_*N*_ can be ignored when the cell is about to fall off. The above forces can be calculated:

20 $${M}_{{F}_{{\rm {B}}}}=\frac{4}{3}\pi {R}^{3}{\rho }_{{\rm {f}}}gL$$

21 $${M}_{{\rm {G}}}=-\frac{4}{3}\pi {R}^{3}{\rho }_{{\rm {c}}}gL$$

22 $${M}_{{F}_{{\rm {N}}}}=0$$

23 $${M}_{{F}_{{\rm {H}}}}=\pi {R}_{{\rm {H}}}^{3}{P}_{{\rm {C}}}$$

24 $$L=\sqrt{({R}^{2}-{R}_{{\rm {H}}}^{2})}$$

where *P*_C_ is the holding pressure, *R*_H_ is the radius of the holding channel. *L* is the distance between point *o* and the center of the cell. Substituting Eqs. (20)–(24), the critical pressure *P*_C_ is

25 $${P}_{{\rm {C}}}=\frac{4}{3}({\rho }_{{\rm {c}}}-{\rho }_{{\rm {f}}}){\left(\frac{R}{{R}_{{\rm {H}}}}\right)}^{3}gL$$

Because of the effect of liquid surface tension^35^, the *P*_C_ is modified as

26 $${P}_{{\rm {C}}}=\frac{4}{3}({\rho }_{{\rm {c}}}-{\rho }_{{\rm {f}}}){\left(\frac{R}{{R}_{{\rm {H}}}}\right)}^{3}gL+\frac{4\sigma \cos \beta }{2{R}_{{\rm {H}}}}$$

where *σ* is the surface tension coefficient of the fluid, and *β* is the contact angle between the fluid and the air. Zebrafish embryos are selected in the experiment. The radius and the density of the oocyte are approximately 500 μm and 2.18 g/cm^3^, respectively. *R*_*H*_ is 375 μm, and *L* is 330.72 μm. So, *θ* is 41.41° with the inverse trigonometric function. The density of the medium is 1.0 g/cm^3^, and *g* is 10.0 m/s^2^. So, *F*_D_ is 3.68 × 10^−6^ *N*, *P*_C_ is 549.93 *Pa*.

## Results and discussion

In this section, multiple experiments are designed to test and demonstrate the performance of the developed system.

### Preparation of experimental study

The day before the experiment, adult male and female fish are placed in breeding tanks with a ratio of 1:2, which is separated by dividers. On the day of the experiment, the dividers in the breeding tanks are removed, allowing male and female fish to come into contact. They usually start to mate and spawn within 10 min. After the zebrafish spawns, the embryos are collected for experiments. The samples are usually injected at the 1-cell–4-cell stage. The experimental vector is PCS2 (vector: tol2-*α*actin-egfp) with a concentration of 150 ng/μL, and each embryo is injected with 2 nL.

The YOLOX and MMPOSE networks are adopted for detecting needle and cell in the field of view, which is employed to determine the spatial relationship between the cell and injector. It is trained from scratch for 300 epochs with a learning rate of 10^−5^. Training took 5 h on a PC with Nvidia GTX 3060, Intel Core i7-7700K CPU, and 16 GB of RAM with PyTorch 1.8. Mean average precision (mAP), and average frame rate per second (FPS) are used to evaluate the performance of neural network algorithms.

### The performance analysis of microinjection system

**Fig. 6:**
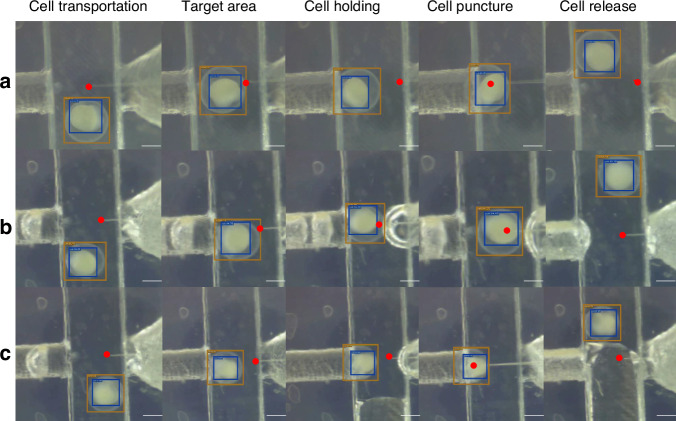
Examples of cell manipulation processes. **a**–**c** Three different cell operation instances. The completed process includes cell transportation, holding, puncture, and release. The YOLOX algorithm was used to detect zebrafish embryos and locate yolk. The MMPOSE algorithm is employed to locate the needle tip, which accurately estimates the position of the tip within the yolk. The scale bar is 500 μm

**Fig. 7:**
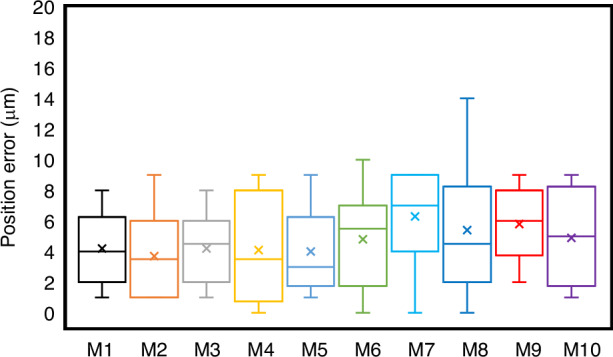
Needle tip positioning error. The 100 images were divided into 10 groups, namely M1, M2,..., M10. The start and end of the boxes denote the first and third quartiles. The band and red dot represent the median, mean, and outliers of the recorded changes, respectively. The maximum and minimum position errors are about 15 and 0 μm, respectively

The complete process of cell puncturing is shown in Fig. 6. When a cell appears within the microscope’s field of view, the algorithm is capable of quickly locating and tracking the cell. The cell is transported under fluid propulsion. When the cell’s centroid is within the vertical coordinate range of the injector, the cell is gripped and positioned. Then, the cell is punctured by the injection, released, and collected into a designated container. Throughout the entire process, the mAP is 95.34%, and the detection speed is 15 FPS (size: 1920 × 1080 pixels). By comparing the manually calibrated position of the needle tip with the detected position of the needle tip in the image, the positioning error of the needle tip is about 4.95 μm, as shown in Fig. 7.

**Fig. 8:**
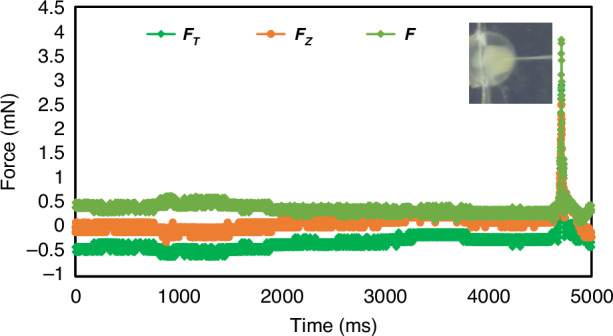
3D mechanical information of cell puncture. *F*_T_ represents the combined force of *F*_*X*_ and *F*_*Y*_. *F* is the total cell force. At the moment of cell puncture, the sensor will undergo a signal jump, which signals that the cell is pierced

Figure 8 depicts the mechanical information of zebrafish embryos during puncture. It is beneficial to the 3D force sensor developed in this research, which is integrated with the microinjector. Before the needle made contact with the cell, the 3D force signal was unchanged. When the needle begins to puncture the cells, the force sensor signal rises sharply. When the cell is punctured, the force sensor signal reaches its maximum and then drops sharply to its initial value. It indicates that the obvious signal jump can be used as the basis for the success of cell puncture. As shown in Fig. 8, the total puncture force of the cell was 3.9 mN, the transverse puncture was 3.1 mN, and the axial force puncture was 2.4 mN.

**Fig. 9:**
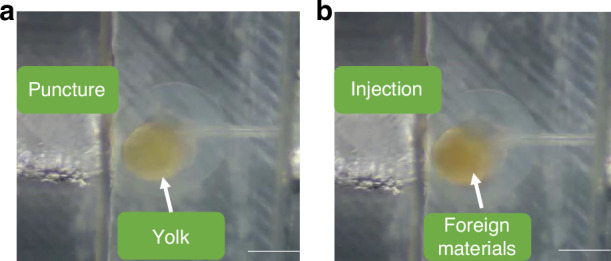
Foreign materials are injected into the cell. **a** The needle is punctured into the yolk. **b** Foreign materials with red marks are injected into the yolk. The scale bar is 500 μm

**Fig. 10:**
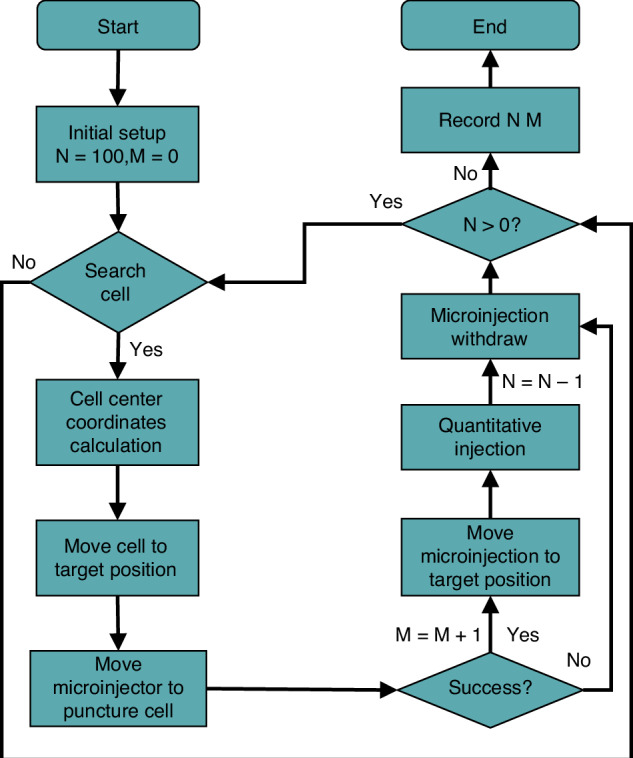
The proposed automatic microinjection manipulation system workflow

The workflow of the proposed automation system is shown in Fig. 10. System initialization parameters *N* and *M*, *N* is the number of cells mainly injected in this task, and *M* is the number of cells successfully pierced. Vision algorithms are used to detect cell and needle positions, and force sensors are used to determine whether the cells have been successfully pierced. This illustration shows that cells successfully injected with PCS2 vector hatch into juvenile fish, and fluorescence signals can be observed after correct expression. An example of zebrafish embryo injection is shown in Fig. 9.

### The performance analysis of batch microinjection

A batch cell injection experiment is designed to test the proposed platform’s performance. Zebrafish embryos of about 1 mm are chosen as the experimental objects. A total of 150 cells were injected in this experiment, of which 50 cells were injected manually and 100 cells were injected by the proposed method. The experimental results are recorded in Table 1. A biocompatible red labeling agent is used to mark whether the cells are successfully punctured. If the injection is successful, the red label is detected. Injection efficiency and cell survival rate are used to evaluate the performance of the designed platform. Injection efficiency *η* is

27 $$\eta =\frac{{T}_{{ {tim}}}}{{N}_{{ {tot}}}}$$

The cell puncture success rate Φ is

28 $${{\Phi}}=\frac{{N}_{{ {puc}}}}{{N}_{{ {tot}}}}\times 100 \%$$

The cell survival rate *N* is

29 $$N=\frac{{N}_{{ {sur}}}}{{N}_{{ {puc}}}}\times 100 \%$$

where *N*_puc_ is the number of cells successfully punctured, *T*_tim_ is the total time to successfully inject *N*_puc_ cells, *N*_tot_ is the total number of cells, and *N*_sur_ is the number of successfully punctured cells that hatch into juvenile fish. The proposed automatic microinjection manipulation system workflow is shown in Fig. 10.

**Table 1:** Comparative analysis of different cell injection methods

Methods	Manual injection	This work
*N*_*tot*_	50	100
*T*_*tim*_ *(s)*	2650	2000
*N*_*puc*_	46	100
*N*_sur_	33	84
*η* *(s/cell)*	53	20
Φ (%)	92	100
*N* (%)	66	84

**Fig. 11:**
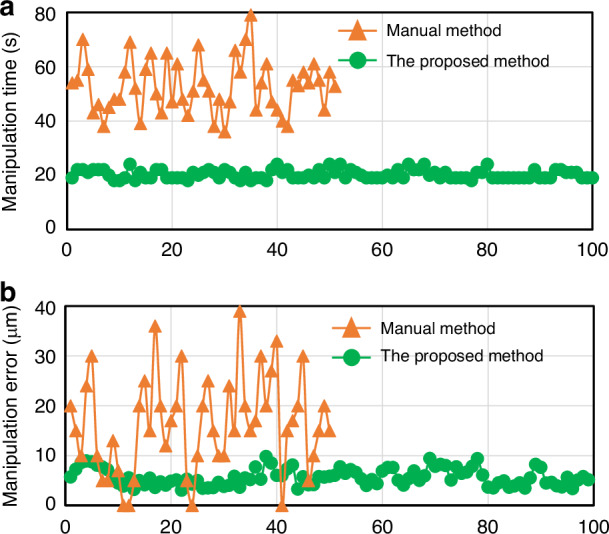
Results of comparison between the manual and proposed methods. **a** The injection time of individual cells. The manipulation time of individual cells is defined as follows: The starting time point of the manual method is to start searching for the cells, and the ending time point is to pull out the injection needle from the cell. The starting time point of the proposed method is that the feeding pump starts to load cells, and the ending time point is that the holding pump completely releases the cells and transports them. **b** Injection position error. The manipulation error is defined as needle tip deviation from the desired position. The actual position of the needle tip is calculated by vision-tracking. The desired position of the needle tip is the yolk center of the cell, which is detected by the deep-learning algorithm

Table 1 shows that the efficiency of manual injection is ~53 s/cell, whereas the efficiency of the proposed method is about 20 s/cell. The proposed method reduces operation time by 33 s compared to manual injection. Manual injection involves frequent switching between high-magnification and low-magnification microscopes to search cells and the injection needle, which takes an additional amount of time. In contrast, the proposed method achieves directed cell transport into the field of view using microfluidic chips and channels, which saves time on cell localization and focusing for subsequent cell operations. The survival rate for the manual injection method and the proposed automatic injection system was 66% and 84%, respectively. The higher performance indicated that the proposed system could significantly improve the survival rate of cells. It is worth noting that we observed that operator efficiency continued to decline with the operation time increased during the experiment (Fig. 11a). It may have something to do with operator fatigue. The disadvantage of artificial puncture is that the location and depth of the puncture are uncertain and depend on the experience and skill of the operator (Fig. 11b). At the same time, the manual method can not guarantee the consistency of puncture. In contrast, the proposed work relies on automated control algorithms to maximize the same location for each cell.

**Fig. 12:**
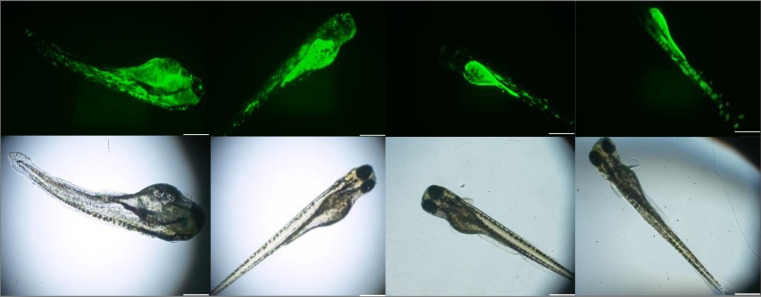
Examples of cells successfully hatching as zebrafish larvae. Observation of zebrafish larvae in visible light mode. Fluorescent signals of zebrafish have been observed under fluorescein isothiocyanate (FITC). The scale bar is 500 μm

Based on the metrics of injection efficiency and survival rate, the proposed automatic injection system is superior to the manual injection approach. Figure 12 shows that the cells injected by the proposed method hatch into zebrafish larvae, and the fluorescence signal is successfully observed under a fluorescence microscope.

### Discussion

In this article, a high-throughput automated system is developed for the injection of batch cells. An automated microinjection system is developed based on a microfluidic chip with a sophisticated structure to transport, hold, and inject cells continuously. Cells do not need to be pre-arrayed and fixed, which saves operation time. The visual perception method based on YOLOX is fused with the developed micro-force sensor to judge whether a cell has been successfully punctured and decide whether to inject foreign material into the cell. A prototype is fabricated to achieve automatic batch microinjection. Experimental results demonstrated that injection efficiency, cell puncture success rate, and cell survival rate are 20 s/cell, 100%, and 84%, respectively. The survival rate of the proposed method increased by 18% compared to the manual injection method. The system can provide a valuable reference for life science research and biological breeding.

Benefiting from the designed microfluidic chip, it can realize the directional transport of cells and transport cells to the microscopic field of view. Traditionally, the cells are placed in the field of vision in advance. The target cells and needle are searched with a low-magnification microscope, and the cell is punctured under a high-magnification microscope. The frequent transformation of the objective lens is one of the factors limiting the improvement of operation efficiency. The efficiency and survival rate of manual operations for cells is 37.08–120 s and 50–70%, which is lower than the performance of robotic operations^24,25^. These are consistent with the conclusions drawn from this research. Literature^29^ demonstrates electroosmotic flow (EOF) transport reagents, which adjust the voltage to control the injection volume. It remains to be verified whether this method has a potential effect on the properties of exogenous substances. There is no such risk with pressure injection.

During microscale automated operations, it is a significant challenge to accurately determine successful cell penetration. Visual perception serves as the primary method to detect cell status based on cell deformation characteristics. However, this is an unreliable source of information. Moreover, excessive cell deformation can lead to irreversible damage to the cells. Therefore, it is significant to develop a microinjector with microforce sensing for batch cell injection. In this research, a compactly structured microinjector with a microforce sensor is developed and integrated into a microfluidic chip. Compared to previous microforce sensors, it can not only sense the cell puncture state with high sensitivity but also inject foreign materials into the cell. The experimental results also prove that the proposed sensor can effectively ensure cell penetration. The survival rate of successfully punctured cells is 84%, which was superior to the result of manual injection. The reason for the unsuccessful incubation of cells possibly be attributed to puncturing critical cell regions. Future research will focus on cell pose adjustment techniques to enhance cell survival rates. The criterion for assessment is based on whether the cells are hatched into fish larvae, rather than measuring cell viability. The experiments demonstrate that this system exhibits good biocompatibility and cell injection capability (Table 2).

**Table 2:** Comparison of different injection methods

Methods	Object	Efficiency (s/cell)	Survival rate (%)	Comment
Pipelined batch-operation^23^	Porcine oocyte	50	–	Unhatched
Microfluidic^28^	HFF	1.7	58.5	Extra time
	VCM		88.4	10 min
Microfluidic^29^	Zebrafish embryos	20	80	EOV
Rotation injection^39^	Zebrafish embryos	>17.3	–	Unhatched
Microfluidic^40^	Zebrafish	41.4	81.42	–
Microfluidic^41^	Zebrafish	18.61	90	–
This work	Zebrafish embryos	20	84	Hatched

*HFF* human foreskin fibroblast, *VCM* vascular cardiomyocyte

The proposed system has functions for cell transport, cell holding, cell puncture, and cell release, which can be used in drug screening and biological breeding research. This achievement can improve research efficiency and cell survival rates, which have practical application potential in biological and medical research. The system provides a paradigm study that can be used not only for zebrafish larvae but also for zebrafish or small organisms (e.g. *Caenorhabditis elegans*), but the microfluidic sizes may be modified to ensure applicability. In the future, we will focus on cell pose rotation technology within microfluidic chips to improve cell survival rates. This is a very challenging task, due to the narrow channels and limited space of microfluidic chips, as well as constraints from the microscope’s field of view. If mechanical contact methods are used to adjust cell pose, it will greatly increase the complexity of system control and operation. Zhang et al.^36^ cleverly utilized microfluidic channels to achieve zebrafish reorientation, while Yuan and Frey^37,38^ systematically summarized the application of physical fields (acoustic fields, magnetic fields, and optical fields) in microfluidic chips for the holding, precise manipulation, and classification of small organisms. These studies provide references and inspiration for our next research. We will combine microfluidic chips with external physical fields to achieve cell posture adjustment within microfluidic chips, which provides the possibility to develop a compact and user-friendly system.

## Conclusion

In this article, a high-throughput automated system is developed for cell microinjection. The sophisticated structure of a microfluidic chip is designed to transport, hold, and inject cells continuously. A deep learning method is employed to detect and locate zebrafish embryos. A microinjector based on microforce feedback has been developed to judge whether a cell has been successfully punctured and decide whether to inject foreign material into the cell. A prototype is fabricated to achieve automatic batch microinjection. Experimental results demonstrated that injection efficiency, cell puncture success rate, and cell survival rate are 20 s/cell, 100%, and 84%, respectively. The survival rate of the proposed method is 18% higher than the manual injection method. This technology could provide an effective solution for various cell-based applications in the life sciences industry.

## Supplementary information

Video

## Data Availability

The data that support the findings of this study are available from the corresponding author upon reasonable request.
